# In situ observation of catalyst nanoparticle sintering resistance on oxide supports via gas phase transmission electron microscopy

**DOI:** 10.1186/s42649-024-00100-4

**Published:** 2024-09-17

**Authors:** Wonjun Kim, Kangsik Kim, Jaejin Kim, Zonghoon Lee

**Affiliations:** 1 Center for Multidimensional Carbon Materials (CMCM), Institute for Basic Science (IBS), Ulsan, 44919 Republic of Korea; 2 Department of Materials Science and Engineering, Ulsan National Institute of Science and Technology (UNIST), Ulsan, 44919 Republic of Korea; 3 Shell International Exploration & Production, Inc, Shell Technology Center Houston, 3333 Hwy 6 S, Houston, TX 77082-3101 USA

**Keywords:** In situ gas phase TEM, Oxide-support, Nanoparticle catalyst, Sintering, MSI effect

## Abstract

Oxide-supported metal catalysts are essential components in industrial processes for catalytic conversion. However, the performance of these catalysts is often compromised in high temperature reaction environments due to sintering effects. Currently, a number of studies are underway with the objective of improving the metal support interaction (MSI) effect in order to enhance sintering resistance by surface modification of the oxide support, including the formation of inhomogeneous defects on the oxide support, the addition of a rare earth element, the use of different facets, encapsulation, and other techniques. The recent developments in in situ gas phase transmission electron microscopy (TEM) have enabled direct observation of the sintering process of NPs in real time. This capability further allows to verify the efficacy of the methods used to tailor the support surface and contributes effectively to improving sintering resistance. Here, we review a few selected studies on how in situ gas phase TEM has been used to prevent the sintering of catalyst NPs on oxide supports.

## Introduction

Metal nanoparticle (NP) catalysts are being used in catalytic converters to reduce the concentration of molecules such as hydrocarbon (C_x_H_y_), nitrogen oxide (NO_x_), and carbon monoxide (CO) present in vehicle and industrial exhaust. These molecules constitute the major sources of gaseous pollutants emanating from many industrial sites, aircraft engines and automobiles (Navarro-Espinoza et al. 2021). Typically, to ensure proper functioning of a catalytic converter in a vehicle, annealing temperatures ranging from 280 ℃ to 650 ℃ under atmospheric pressure conditions is required for vehicles to work properly with the desired chemical reaction (Laskar et al. 2021). The surface of metal NP catalysts has specific active sites that are essential in the catalytic conversion of gaseous pollutants. In general, it is considered to be advantageous to reduce the size of NPs to enhance catalytic efficiency by increasing the number of active sites. However, when metal NP catalysts are exposed to high temperatures, also known as “Tammann temperature,” they gain sufficient mobility to diffuse on the support surface (Dai et al. 2018). Under these conditions, the atoms in the metal NPs escape from their interatomic binding in the catalyst and adhere to the surface of other adjacent NPs to begin the process of sintering.

There are two representative mechanisms to explain the sintering of NPs at high temperatures. According to the first, the disappearance of relatively small-sized NPs occurs, allowing only large NPs to survive, known as “Ostwald Ripening (OR).” The second mechanism called “Particle Migration and Coalescence (PMC),” is operative when NPs migrate on the surface by breaking their bonding from the support, followed by merging with adjacent NPs to increase in size (Hansen et al. 2013; Simonsen et al. 2011). In both cases, sintering occurred at high temperatures causes NPs to coalesce, which is detrimental to their catalytic performance (Yang et al. 2008). The principal method to reduce the sintering of metal NPs is to place them on a support made of an oxide such as TiO_2_, ZrO_2_, CeO_2_, or MnO_2_. These supports are not only less prone to sintering than bulk metal NPs, but also have excellent mechanical properties, thermal stability, and corrosion resistance (Bagheri et al. 2014; Busca 2014). Additionally, it is possible to improve catalytic performance and the metal support interaction (MSI) effect by introducing surface modifications (Yang et al. 2017). The MSI effect can be explained as the bonding of NPs to a modified support surface to improve their sintering resistance due to this modification (Lou et al. 2020).

It has been widely reported that modifying the surface layer of the oxide support can prevent sintering and improve catalytic performance. However, a direct verification of the theoretical models for OR and PMC, which are the two main mechanisms of sintering, is currently lacking. In this context, images with high spatial resolution to record the immediate response of NPs under operating conditions are highly desirable (Hwang et al. 2020). Experimental investigations confirming the effective control of NPs sintering have been also reported. The sintering resistance of NPs has been elucidated through X-ray absorption spectroscopy (XAS) measurements. This method facilitates the characterization of bonding between the catalyst NPs and the oxide support, thereby establishing a direct association with an enhanced MSI effect. (Nagai et al. 2011). This characterization technique provides evidence for the improved sintering resistance due to the increased binding between the metal NP and the support. Using TEM, the size distribution of NPs is determined from multiple images taken at various operating conditions (Wang et al. 2017; Yentekakis et al. 2016).

However, there is still insufficient experimental evidence that allows to unambiguously identify sintering mechanisms such as OR and PMC. In situ scanning tunneling microscopy (STM) allows to directly observe the process of sintering in response to temperature changes (Lu et al. 2007). This method offers the advantage of acquiring data during the intermediate stages of sintering. However, the main limitation of STM is that it can only be used to observe conductive materials. Consequently, the development of optimal methodologies for the identification of sintering mechanisms under realistic reaction conditions represents a significant area of interest. In situ gas phase TEM has been developed as a method for the direct observation of the sintering behavior of NP catalysts. Previous works have approached this subject by inducing sintering inside a TEM under the conditions of reaction by gradually raising the temperature to over 1000 ℃ (DeLaRiva et al. 2013). The use of in situ gas phase TEM has permitted the investigation of the various methods by which sintering can be prevented in order to enhance the efficacy of the NP catalyst. For example, confirming the change in size and number of NPs is an easy way to quantify sintering resistance. In this review, we first present the in situ gas phase TEM system as a means to study catalyst sintering studies. Next, we describe several experimental examples where this technique has been effectively used to evaluate the effectiveness of the different surface modifications of the oxide support. These studies have presented ways to improve sintering resistance of a catalyst during annealing at high temperatures under atmospheric pressure conditions. The studies selected in this review have been classified depending on the methods of surface treatment applied to improve the sintering resistance of the catalyst.

## Two types of in situ gas phase TEM techniques

**Fig. 1:**
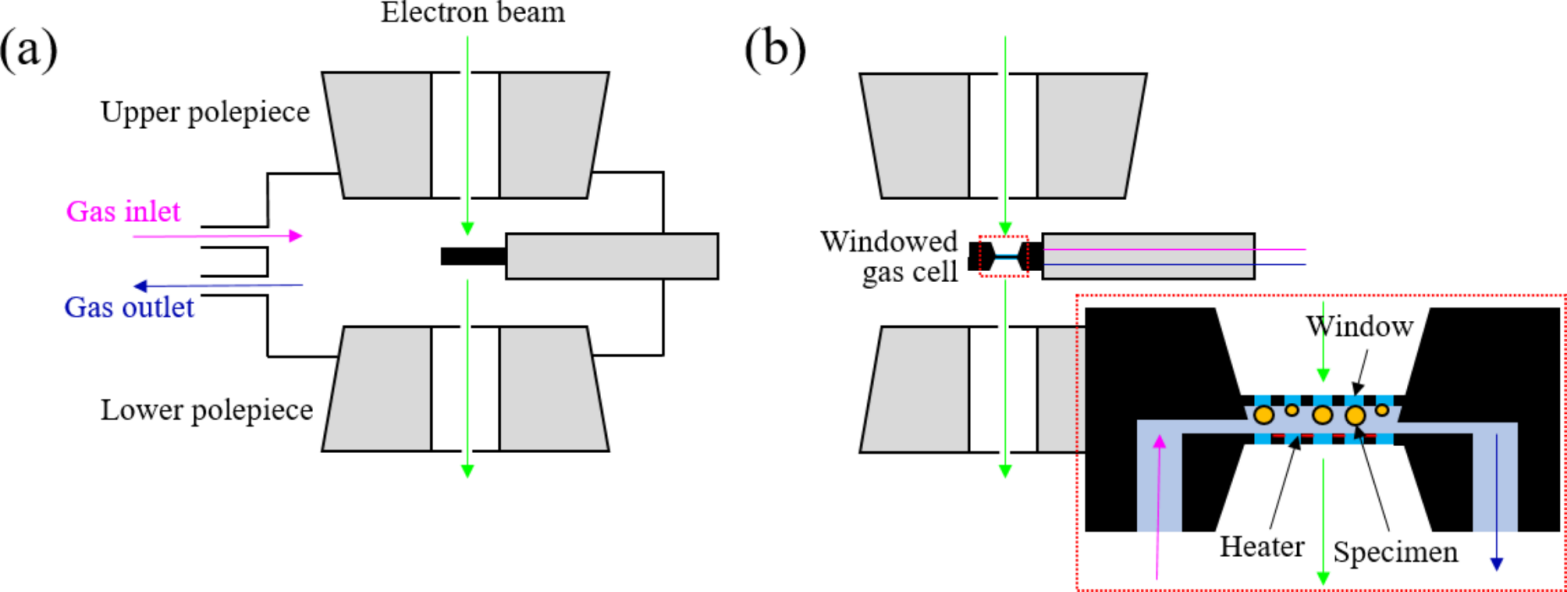
Schematics of the two types of in situ gas phase TEM techniques. (**a**) Environmental TEM (ETEM) between the two polepieces of the objective lens. (**b**) Windowed gas cell between the two polepieces of objective lens in a conventional TEM as well as an enlarged view of the cross section of the windowed gas cell

The development of the in situ gas phase TEM techniques has enabled to obtain direct experimental evidence to support the theory that NP sintering is closely related to catalytic performance. The main capability of in situ gas phase TEM in catalyst studies is to reproduce the sintering behavior of NP catalysts under atmospheric pressure conditions to simulate the actual reactions. It is very challenging to exactly replicate conditions of catalyst sintering within a TEM, which requires a vacuum environment. In situ gas phase TEM circumvents this difficulty by maintaining high vacuum inside the column to preserve the beam path while simultaneously enabling the sample to be studied under atmospheric pressure conditions (Fan et al. 2016). Usually, two types of in situ gas phase TEM techniques are used in catalyst sintering studies. One is the environmental TEM (ETEM) and the other is the windowed gas cell. Both types can simultaneously provide heat and gas flow to the supported NP catalyst sample. Commonly, these in situ gas phase TEM techniques can heat the specimen using a resistance heating method and the temperature can be monitored with a thermocouple integrated inside the holder. However, a limitation of this method is an accurate measurement of the temperature in a specimen in TEM due to the factors such as thermal connection, specimen composition, thermal conductivity of the supporting substrate, the gas pressure, etc. Therefore, it is essential to calibration the local temperature accurately (Winterstein et al. 2015). However, the main difference between the two approaches lies in the manner of creating a gas environment. In the former, modification to the structure of the TEM column generates the necessary conditions, whereas in the latter, a gas line is inserted into the specimen holder. The two configurations are shown in Fig. 1(a) and (b).

In the case of the ETEM, a gas is injected into the space between the upper and lower polepiece, where a reaction environment is created. Although this space is not completely sealed due to the small orifices that allow the electron beam to pass through, additional pumps are attached to each polepiece space to prevent gas leakage (De et al. 2023). A holder with a heating chip loaded with the sample containing the catalyst NPs is placed in the path of the electron beam. One important feature of this setup is that there is no need to use special holders, such as a gas flow line as for the windowed gas cell. This implies that ordinary TEM holders can be used instead. Moreover, as compared to the windowed gas cell, there are no obstacles such as a SiN_x_ window in the beam path that can cause beam scattering and limit spatial resolution. However, the maximum gas pressure in the ETEM is usually limited to approximately 100 Torr, which is lower than the atmospheric pressure (Tanaka et al. 2020). Since the space of TEM column occupied by the gas is large, elevating the gas pressure increases the possibility of beam scattering. Hence, the interaction of the e-beam with gas molecules affects the spatial resolution (Ye et al. 2020). For this reason, it cannot be asserted that catalyst studies conducted in the ETEM replicate the real conditions found in industrial production site.

In the case of the windowed gas cell, a specially designed holder is employed. This holder is structured to form a cell with two silicon Micro Electro Mechanical Systems (MEMS) chip surfaces integrating transparent SiN_x_ windows. The specimen is sandwiched between two electron-transparent windows, effectively sealing the gas atmosphere from the high vacuum of the TEM column. This configuration ensures that the injected gases circulate exclusively within the cell and do not leak to other areas. The schematic in Fig. 1(b) shows the holder containing the windowed gas cell located between two polepieces of the objective lens. Unlike ETEM, the injected gas enters and exits along a designated line inside the holder, which means that there is no need to block the area of ​​the objective lens from other area inside the column to prevent gas leakage. The electron beam transmitted through the window forms an image of the sample located within the space between two windows in the gas environment. One of the main advantages in this set up is that the windowed gas cell does not require an additional pump in the TEM column unlike the ETEM. Also, gas pressures close to ambient pressure (Creemer et al. 2008) are attained, which allows to replicate the actual reaction environment used in industrial sites. Hence, sintering resistance of catalyst NPs can be precisely determined. However, when the electron beam passes through the SiN_x_ windows, it causes beam scattering, reducing spatial resolution. Moreover, the gases flowing inside the holder cause a bulging effect on the SiN_x_ windows, which increases the width of the space between two windows. This causes the electron beam to collide with more gas molecules, resulting in increased scattering, which further reduces spatial resolution (Ye et al. 2020). Moreover, the loading of the sample onto the SiN_x_ window and the subsequent alignment of the two windows require a high degree of precision and expertise (De et al. 2023).

## Methods to study sintering resistance with in situ gas phase TEM

In general, direct observation on the structural modification of NPs is essential for confirming the improved sintering resistance. However, it is challenging to make a clear size-dependent observation in a gas environment using other equipment, such as conventional microscopy and X-ray techniques. This section describes the different research studies in which the surface of the support material is modified to improve the sintering resistance of dispersed NPs. It also introduces representative studies on surface modification that utilized the specific equipment as to extract the size-dependent results. In particular, both ETEM and windowed gas cell have employed extensively to examine the catalyst NPs on the support material surfaces throughout the reaction and to assess the sintering resistance at elevated temperatures under atmospheric pressure conditions.

### Inhomogeneous defects on oxide support

**Fig. 2:**
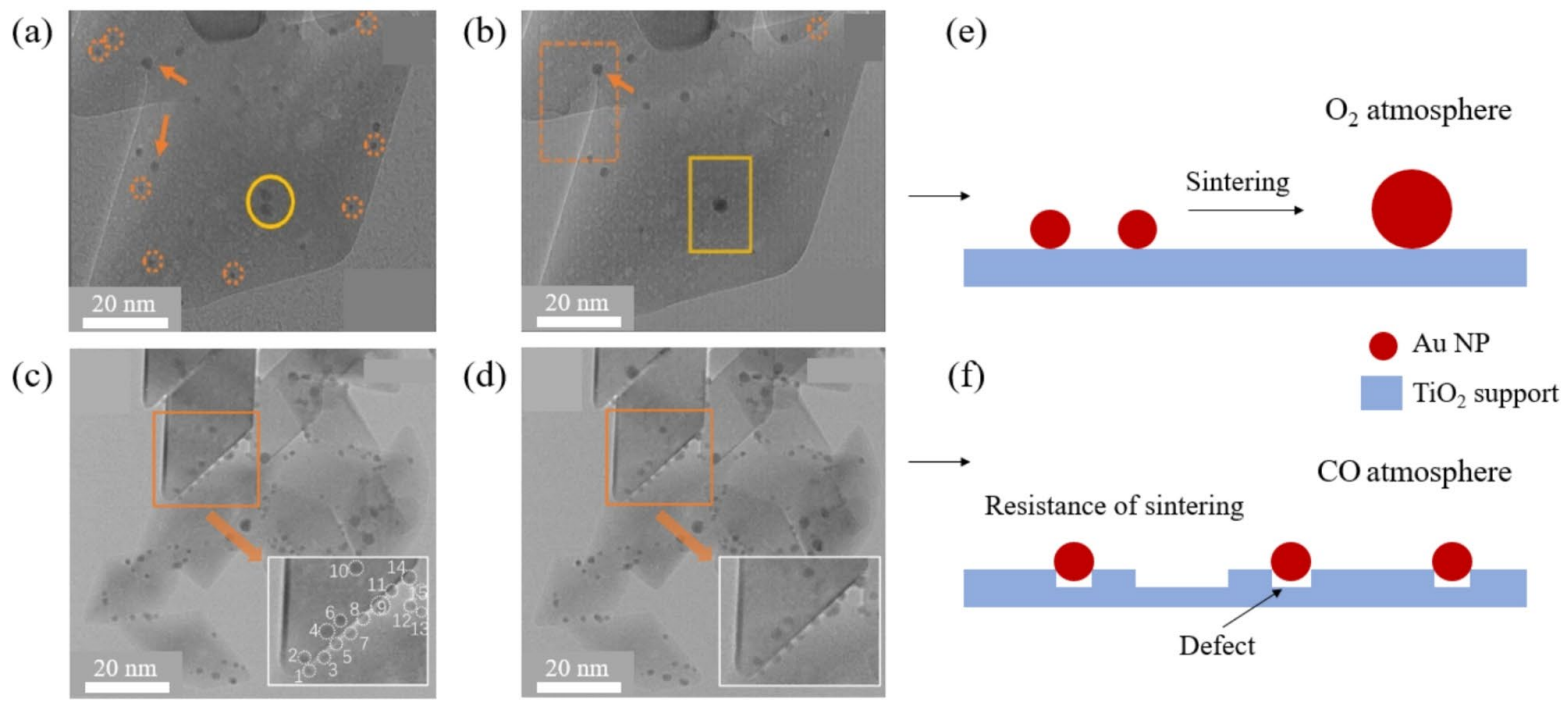
(**a**) and (**b**) In situ ETEM images showing the sintering behavior of Au-TiO_2_ catalysts in oxygen atmosphere (0.05 Pa O_2_ at 700 °C). The ETEM images were acquired at 0 and 60 min, respectively. To direct the readers’ attention, NPs that were sintered via OR are indicated by dashed circles, while NPs that were sintered via PMC are indicated by solid circles. The orange arrows indicate the location of NPs that grew via OR. Schematic of Au NPs’ sintering in O_2_ atmosphere. (**c**) and (**d**) In situ ETEM images showing Au-TiO_2_ catalysts in CO atmosphere (0.05 Pa CO at 700 °C) taken at 0 and 60 min, respectively. Insets are enlarged images of the area marked by the orange rectangles. (**e**) and (**f**) Schematic of Au NPs sintering in O_2_ and CO atmosphere. Scale bar, 20 nm for (**a**)-(**d**), Reprinted from Li et al. (2020) (Journal of Catalysis 388, 84–90) with the permission from the Elsevier

Previous studies using Density Functional Theory (DFT) calculation have reported that steps and defect sites on the support surface play an important role in the MSI effect of the catalyst NPs (Gong et al. 2008; Wan et al. 2018). It has been also reported that the presence of defects on the support surface hinders the migration of NPs even at high temperatures. These inhomogeneous defects, ranging from vacancies to surface steps, effectively reduce catalyst sintering on the support by preventing the movement of NPs by anchoring them on the surface (Belgamwar et al. 2023; Cai et al. 2022). An in situ study with gas phase TEM to directly identify the contribution of surface defects in enhancing sintering resistance has been reported (Li et al. 2020). Figure 2 shows the structural evolution of Au NPs on the surface layer of a TiO_2_ support as observed using ETEM. Sintering was confirmed by tracking specific Au NPs during long-term observation and recording changes in their size, number and distance to other NPs. This enabled the investigation of OR and PMC, known to be the major sintering mechanisms. As shown in Fig. 2(a) and (b), the sizes of a few NPs increased during annealing in O_2_ atmosphere which was indicative of OR, while the rest of the particles disappeared. The coalescence of two adjacent NPs into one larger NP was also observed, indicating the simultaneous presence of PMC. These results clearly proved that sintering of the NPs occurred. An opposite result was observed for supported Au NPs when annealed under the same conditions in CO atmosphere as shown in Fig. 2(c) and (d). In contrast to O_2_ atmosphere, no notable change in the number and size of NPs was observed, indicating that annealing in CO atmosphere does not lead to sintering. This difference has been attributed to the injected gas affecting the surface layer of the TiO_2_ support. Annealing under CO atmosphere induced inhomogeneous defects such as voids and steps on the surface layer of the TiO_2_ support, in contrast to annealing under O_2_ atmosphere, where this effect is absent. The presence of such defects strengthened the binding between catalyst NPs and the oxide support, with Metal NP atoms occupying defect sites such as vacancies. Theoretical calculations have shown that the interaction energy between the NP atoms and the defect has a negative value, indicating that the NPs bind preferentially to defect sites (Matthey et al. 2007; Rieboldt et al. 2014; Sanchez et al. 1987). Thus, annealing under CO atmosphere can create surface defects which can roughen the TiO_2_ support surface and these surface defects play a key role in preventing the sintering of supported Au NPs.

### Adding a rare earth element into the oxide support

**Fig. 3:**
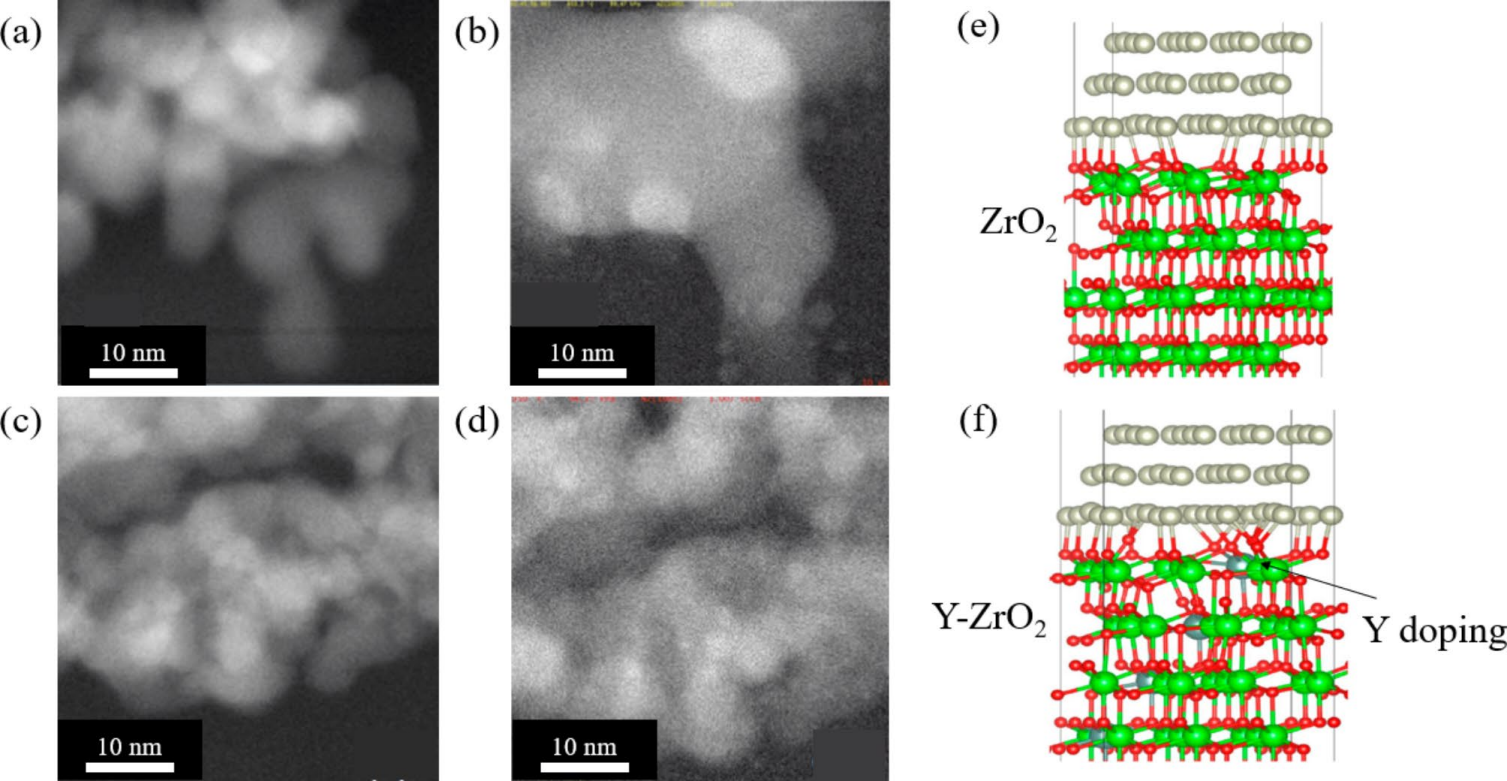
In situ gas phase STEM images of Rh/ZrO_2_ at (**a**) 300 ℃ and (**b**) 950 ℃ in N_2_ atmosphere. (**c**) Side view of the interface between a three-layered Rh (111) structure and the ZrO_2_ support. In situ gas phase STEM images of Rh/Y–ZrO_2_ at (**d**) 300 ℃, and (**e**) 950 ℃ in N_2_ atmosphere. Reprinted from Nakayama et al. (2021) (Catalysts 11) with permission from MDPI

Studies have shown that introducing a rare earth element into the oxide support effectively improves the MSI effect between the catalyst NP. One study demonstrated that the addition of a rare earth element such as Nd can improved the MSI effect between Rh NPs and a La–ZrO_2_ support (Tanabe et al. 2012). H_2_-temperature-programmed reduction (TPR) and Extended X-ray Absorption Fine Structure (EXAFS) measurement pointed to increased interaction between the supported Rh NPs and the Nd_2_O_3_ enriched-surface of the La–ZrO_2_ support. The Rh–O–Nd binding formed during the oxidation allowed a better dispersion of Rh NPs. The MSI effect was maintained during the redox aging test at high temperatures. These observations permit the speculation that the sintering resistance of Rh NPs on Nd_2_O_3_ surfaces is caused by an anchoring effect from the strong Rh–O–Nd bond. In another work, in situ gas phase TEM was used to study the sintering of Rh NPs on the surface layer of ZrO_2_ and Y doped ZrO_2_ support (Y–ZrO_2_) (Nakayama et al. 2021). Figure 3(a) and (b) show the process in which the size and number of Rh NPs on the surface layer of the ZrO_2_ support change as the temperature is increased under N_2_ and O_2_ atmospheres, as observed by in situ gas phase STEM with a windowed gas cell. The sintering of Rh NPs starts at 900 ℃, and as the temperature is increased, the size of the Rh NPs increases proportionally, as verified by measuring changes in diameter of NPs from in situ gas phase STEM images. However, on Y–ZrO_2_ support, under the same conditions, no change in the size of NPs is observed as shown in Fig. 3 (c) and (d). Moreover, at the end of the annealing process, the sizes of the Rh NPs on Y–ZrO_2_ were found to be smaller compared to those on ZrO_2_. Results of DFT calculation including side view schematics of the interface between Rh NP and ZrO_2_ and Y–ZrO_2_ support materials show in detail, the binding of the Rh NP to the support surface layer in Fig. 3 (e) and (f). It can be seen that the number of bonds between the atoms constituting the Rh NP to oxygen atoms is higher around the Y doped region. Additionally, DFT calculations showed that the binding strength between Rh NPs on Y–ZrO_2_ support is much stronger than that observed on the ZrO_2_ support. The increased MSI effect of the ZrO_2_ support due to Y doping has been explained from the standpoint of ionic conductivity. Pure ZrO_2_ is an insulator and has no ionic conductivity, whereas in the case of Y–ZrO_2_, a back-spillover of the ionic species is induced as the temperature is increased. This causes multiple oxygen atoms on the support surface to move towards the NP’s surface, which contributes to increasing the binding strength between NPs and the support (Dole et al. 2013; Isaifan et al. 2015).

### Facet–dependent catalyst sintering

The facet of the support surface layer has a significant influence on the MSI effect between the supported NPs and the oxide support, which in turn impacts both the sintering effect and the efficiency of catalytic performance for reactions including CO oxidation (Spezzati et al. 2019). Theoretically, facet-dependent sintering behavior has been explained in terms of the difference in diffusion barrier depending on the facet of the surface layer (Mehta et al. 2017). Depending on the type of facet, the number of dangling bonds on the support surface is different. Results from DFT calculations have shown that a particular facet with a large number of dangling bonds has a high surface energy. Consequently, this leads the creation of a high diffusion barrier between NPs and the support surface (Wan et al. 2019). This conclusion has been supported by experimental results obtained by in situ gas phase TEM, where the nature of the facet has a significant impact on the sintering resistance of the supported NPs. For example, the sintering behavior of supported Au NPs on TiO_2_ surfaces observed using in situ ETEM has been reported (Yuan et al. 2018). TiO_2_ supports with two different facet types viz., the (101) and the (001) surfaces, were prepared. Sintering behavior was confirmed by the size of NP and the distance between the NPs. At the (101) surface, the sintering of Au NPs coupled with OR and PMC was confirmed by measuring the decrease in size of a selected NP and its coalescence at 500 ℃ under O_2_ atmosphere. In contrast, on the (001) surface, no sintering was detected under the same conditions. An in situ gas phase TEM study on the influence of the facet type of the surface layer of a ZnO support during sintering of supported Pd NP has also been reported (Pu et al. 2021).

**Fig. 4:**
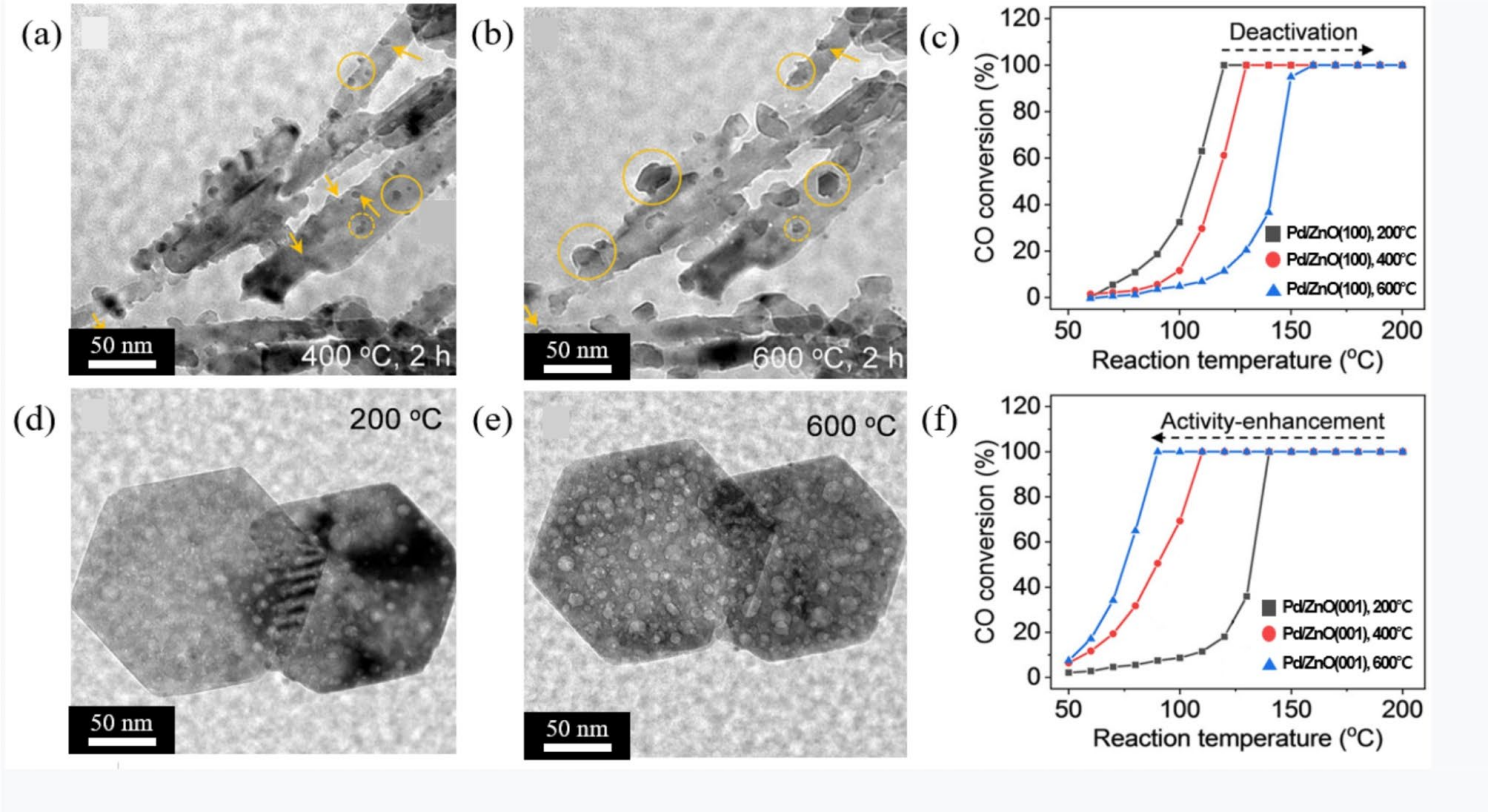
(**a**) and (**b**) In situ gas phase TEM images were obtained to investigate the sintering behavior of NPs on facet of ZnO nanorods with (100) at 200 and 600 °C in air at atmospheric pressure. The images demonstrated that NPs that sintered via PMC and OR exhibited distinct morphologies. The NPs that sintered via PMC were observed to form smaller, more uniform particles, while those sintered via OR exhibited severe aggregation. (**c**) Light-off curves of CO oxidation reactions over Pd NPs on facet of ZnO nanorods with (100) catalysts after calcination at 200, 400, and 600 ℃ in the air. (**d**) and (**e**) In situ gas phase TEM images of Pd/ZnO nanosheets with (001) at 200 and 600 °C in the air, respectively. (**f**) Light-off curves of CO oxidation reactions over Pd NPs on ZnO nanosheets (001) catalysts after calcination at 200, 400, and 600 ℃ in the air. Scale bar, 50 nm for (**a**), (**b**), (**d**), and (**e**). Reprinted from Pu et al. (2021) (The Journal of Physical Chemistry C 125, 20351–20359) with the permission of the American Chemical Society

Figure 4(a) and (b) show in situ gas phase TEM images of Pd NPs on the surfaces of ZnO supports with two kinds of facets having different crystal orientation annealed in air at temperatures ranging from 200 to 600 ℃. The sintering behavior of Pd NPs on facet of ZnO nanorod with (100) was confirmed by a decrease in the number of NPs and an increase in the size of a few NPs. In contrast, no sintering was observed for Pd NPs on facet of ZnO nanosheet with (001) under the same experimental conditions as shown in Fig. 4(d) and (e). This different behavior demonstrates that the crystal orientation of surface layer contributes to enhancing the MSI effect between the supported NPs and the surface layer (Pu et al. 2021). These experiments also reveal the impact of sintering resistance on the efficiency of catalytic performance. Figure 4(c) and (f) show the light-off curves showing the CO oxidation efficiency of Pd NPs on the surfaces of ZnO nanorods and nanosheets, respectively. While increasing the calcination temperature, the CO conversion rate decreased more significantly for Pd NPs supported on the (100) facet of ZnO nanorods in Fig. 4(c) compared to Pd NPs on the (001) facet of ZnO nanosheets in Fig. 4(f). These results indicate that deactivation of catalyst NPs due to sintering can be prevented by selecting the appropriate crystal orientation.

### Encapsulation

Encapsulation is known to be as one of effective strategies for preventing sintering and maintain catalytic performance by increasing the MSI effect between the oxide support and catalyst NPs (Das et al. 2018; Liu et al. 2021; Masoud et al. 2022). In recent times, in situ gas phase TEM investigations have contributed to obtaining an in-depth understanding of the relationship between encapsulation and the MSI effect. In a particular study, Pd NPs encapsulated by a TiO_2_ layer were annealed in the redox atmosphere. When annealed at 250 ℃ under the reducing atmosphere (H_2_(5 vol %)/Ar, 1 atm), amorphous TiO_x_ layer begins to form around the surface of the Pd NPs (Zhang et al. 2016). The TiO_x_ layer then starts to spread from the interface to surround the Pd NP and the support. The amorphous TiO_x_ surrounding the Pd NP starts to crystallize at 500 °C, forming the thin layer which develops into an epitaxially grown encapsulation layer on the Pd (111) crystal plane. In addition, the crystallization of encapsulation layer that grows epitaxially on the surfaces of NPs may degrade catalytic activity. After the occurrence of the SMSI effect by the presence of oxide overlayer on the NP’s surface, the access of gas molecules to the active sites of the catalyst is blocked. It has been reported that epitaxial interaction between Au and ZnO showed degradation of CO oxidation rate. (Liu et al. 2012). The presence of an epitaxial layer was confirmed through high-angle annular dark-field scanning transmission electron microscopy (HAADF STEM) and electron energy loss spectroscopy (EELS).

In case of the encapsulating with oxide layer, it has a wider contact area between NPs and the oxide layer than the case of MSI effect as we mentioned above. This phenomenon is often referred to as ‘strong metal support interaction (SMSI)’ due to their capabilities to enhance catalytic reaction and improve thermal stability. For example, the SMSI effect mostly occurs in reduction atmosphere and the encapsulating Ti species exhibits a low oxidation state, the metal–Ti^3+^ interaction was proposed as the driving force for the encapsulation process. Besides, such encapsulation process occurred for TiO_2_-supported metals with high surface energy, such as Pt, Fe, Pd, and Rh, but not with metals with low surface energy such as Au. And thus proposed the surface energy minimization as one of the dominant driving forces for encapsulation (Li et al. 2022). It has been reported that the method creates encapsulating layer, so that the NPs are strongly stabilized on support preventing detachment from the support surface in high temperature (Kaiser et al. 2023).

In addition to methods that create an oxide shell, there are other encapsulation method by using organic layers. Similar to oxide layers, the organic layers encapsulating NPs contributes to isolate the catalytically active NPs, thereby preventing sintering at high temperatures while maintaining catalytic performance. Furthermore, this method can also lead to decomposition of the catalyst NPs. As a result, the size, shape and composition of nanoparticles after heating process could be different from those of pristine NPs. (Joo et al. 2009). Such organic layer can resist sintering at high temperatures and recover the activity of sintered and deactivated catalysts at the same time, whereas the oxide shell has a side effect to interfere contact between the surfaces of NPs and reaction gas molecules which degrades catalytic performance.

**Fig. 5:**
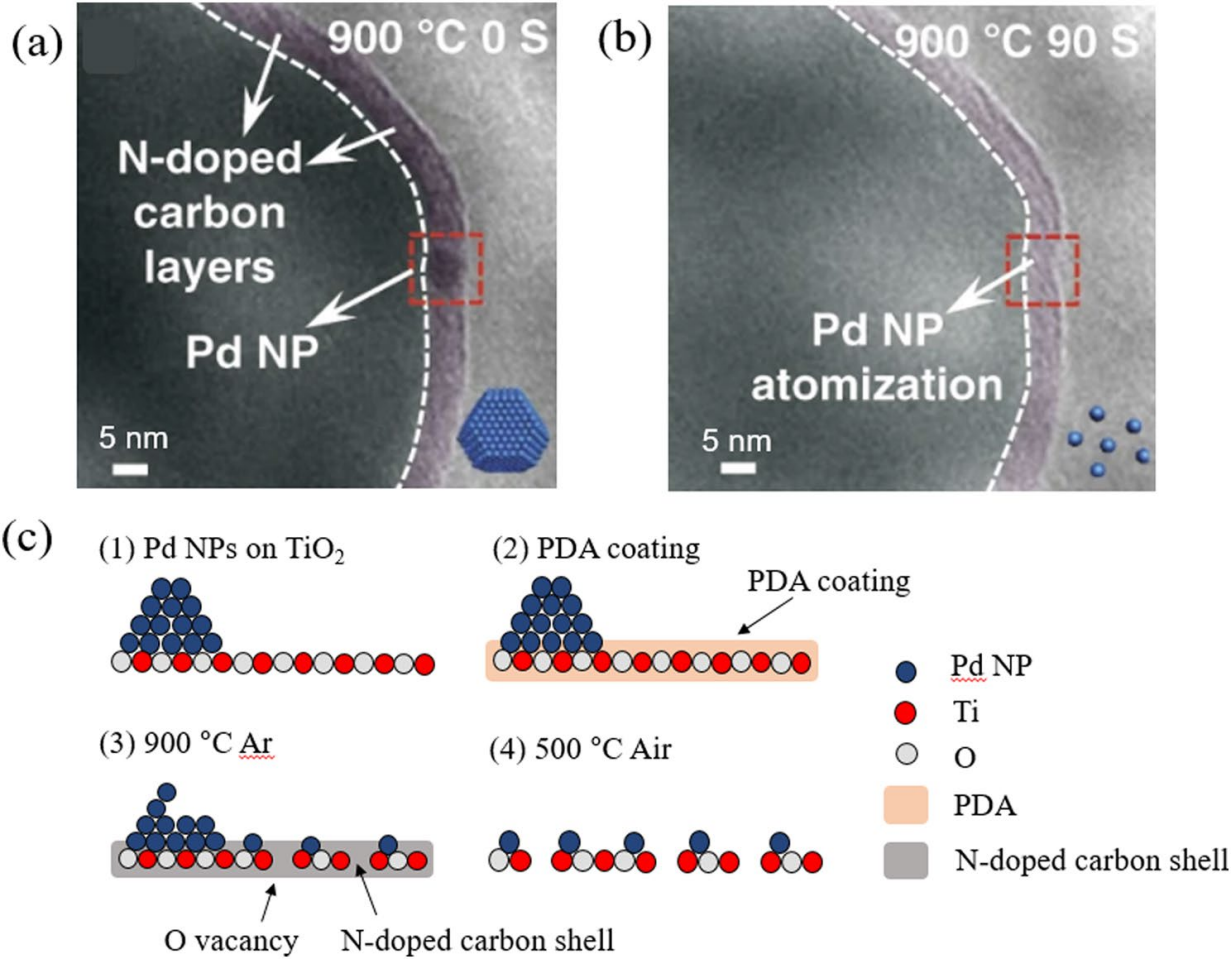
(**a**) and (**b**) Representative TEM images of Pd NPs/TiO_2_/C acquired at 900 °C at different times with TEM under Ar atmosphere. (**c**) Schematic illustrations of the N-doped carbon with atomization of Pd NPs/TiO_2_, which results in Pd single atoms/TiO_2_. Reprinted from Zhou et al. (2020) (Nature Communications 11, 335) with the permission of the Nature Communications

One of the studies utilizing this method is using Polydopamine (PDA) coating method to TiO_2_ surface with Pd NPs. In addition, formation of oxygen vacancies on the oxide support during the heating process in Ar environment reduces mobility of the NPs by anchoring them. This encapsulation method combines with surface modification of defect formation. First of all, TiO_2_ support surface is coated with PDA. During the 900 °C heating in Ar environment, PDA layer transforms into N-doped carbon layer that encapsulates the Pd single atoms dispersed from the Pd NPs. At the same time, oxygen vacancies on TiO_2_ surface were formed. As we previously mentioned in the Sects. 3 − 1, these vacancies act as the spaces where the dispersed Pd single atoms which were following Ostwald ripening principle can be anchored. Finally, after heating process in air, the carbon layer is removed so the captured Pd single atoms can be directly located on the vacancies of TiO_2_ surface. The changes in the size and number of NPs were tracked by in situ gas phase TEM with a windowed gas cell (Zhou et al. 2020). In Fig. 5(a) and (b), the dispersion of sintered Pd NPs in the presence of a shell of N-doped carbon shell formed on the surface layer of the TiO_2_ support is shown. By following the decrease in contrast of the Pd NPs, the dispersion of NPs on the atomic scale was confirmed. Figure 5(c) shows the schematics of the mechanism by which the N-doped carbon shell on the TiO_2_ support induces the dispersion of Pd NPs. High-temperature annealing (900 ℃, 90 s) under Ar atmosphere inside the windowed gas cell induces a transformation of PDA into N-doped carbon layers accompanied by the creation of oxygen defects on the surface of the TiO_2_ support. The N-doped carbon shell thus plays a crucial role in inhibiting sintering of Pd NPs during annealing. Also, multiple N defects inside the carbon shell trap the Pd atoms that are expelled from the Pd NPs through thermal diffusion. The N-doped carbon shell is removed by annealing at 500 °C in the air. The dispersed Pd atoms are then trapped by oxygen defects present on the surface of the TiO_2_ support. The deactivation of the catalyst NPs due to the encapsulation can be avoided by removing the N-doped carbon shell after the dispersion step. In another experiment, TiO_2_ with three facets were prepared and the MSI effect between the supported Pd NPs and the support material was compared for the three cases (Tang et al. 2021). TiO_2_ supports with (101), (100), and (001) facets were used in the experiment. The structural evolution of the Pd NP and that of the TiO_2_ support were observed in real-time gas phase TEM under oxidizing conditions (O_2_ (20 vol%)/ N_2_ at 1 atm) during annealing. It was confirmed that as the temperature was increased to the annealing temperature (550 ℃ for 60 min), Pd NPs were encapsulated on the (101) and (100) faceted TiO_2_ surfaces. In contrast, no encapsulation of Pd was observed for the TiO_2_ with the (001) facet under the same conditions. This experiment demonstrates that in situ gas phase TEM provides an unprecedented opportunity to investigate how the encapsulating layer was formed and how it contributed to the MSI effect, to thereby modify the sintering behavior.

## Conclusion

We have described various experimental techniques used to elucidate how enhanced sintering resistance can be achieved by manipulating the surface layer of the oxide support to enhance the MSI effect. Our emphasis has been on utilizing in situ gas phase TEM to investigate the real time sintering process of NP catalyst. Surface defects strongly influence the change in the size and number of NPs and also affect the rate of coalescence in the high temperature gas environment. The MSI effect between the NP and the support is improved by doping the support material with a rare earth metal, since there was no change in the size of the NPs even after annealing at high temperatures under atmospheric conditions. Furthermore, the dependence of the rate of sintering on the facet type of the support was measured by imaging the changes in the size and number of NPs using in situ gas phase TEM. These results were then correlated with experiments to compare the catalytic performance according to the support facet. Encapsulation was found to prevent NP sintering and resulted in single-atom dispersion of NPs; the different experiments described in this review are listed in Table 1. Thus, in situ gas phase TEM can be used to validate the processes of OR and PMC, two prevalent theoretically proposed mechanisms of sintering. This review demonstrates the powerful experimental technique of in situ gas phase TEM that serves as a bridge between theory and experimental observation. Using this technique also allows to establish the negative effect of catalyst NPs sintering on their catalytic performance. Such results are also expected to provide important guidelines on how to improve sintering resistance of supported catalyst NPs.

**Table 1:** List of sintering resistance studies including in situ gas phase TEM observation

Holder type	NP	Support	Sintering resistance method	Reference
ETEM	Au	TiO_2_	Surface defect formation.	Li et al. (2020)
			Facet modification of support surface	Yuan et al. 2018
	Pd		Support encapsulation rate depends on facet	Tang et al. 2021
Windowed gas cell	Pd	TiO_2_	NP encapsulation with PDA	Zhou et al. (2020)
			Amorphous TiO_x_ layer encapsulation	Zhang et al. 2016
		ZnO	Facet modification of support surface	Pu et al. (2021)
	Rh	ZrO_2_	Adding rare earth (Y) element in support	Nakayama et al. (2021)

## Data Availability

Not applicable.

## Declarations

Zonghoon Lee is the Editor-in-Chief of Applied Microscopy. The status of Editor-in-Chief has no bearing on editorial considerations.

## Abbreviations

DFT: Density Functional Theory
EELS: Electron Energy Loss Spectroscopy
ETEM: Environmental Transmission Electron Microscopy
EXAFS: Extended X-ray Absorption Fine Structure
HAADF STEM: High-angle Annular Dark-field Scanning Transmission Electron Microscopy
MEMS: Micro Electro Mechanical Systems
NP: Nanoparticle
OR: Ostwald Ripening
PDA: Polydopamine
PMC: Particle Migration and Coalescence
STM: Scanning Tunneling Microscopy
TEM: Transmission Electron Microscopy
TPR: Temperature Programmed Reduction
XAS: X-ray Absorption Spectroscopy
